# Nonobstructive Recurrent Cholangitis After Hepatobiliary Tract Diversion in Patients With Surgically Altered Anatomy: A Challenging Problem

**DOI:** 10.14309/crj.0000000000001639

**Published:** 2025-03-05

**Authors:** Rahul Karna

**Affiliations:** 1 Division of Gastroenterology, Hepatology & Nutrition, University of Minnesota, Minneapolis, MN

In the recent article “Successful Use of Fecal Microbiota Transplantation in Management of Nonobstructive Recurrent Cholangitis Following Total Pancreatectomy and Islet Auto-transplant” by Scott et al,^1^ the authors demonstrated successful resolution of recurrent cholangitis without any obvious discernible obstructive etiology by intestinal microbiota transplant (IMT).

Hepatobiliary or pancreatic surgeries including total pancreatectomy with islet autotransplant often involve biliary tract diversion and creation of biliary-enteric anastomosis (BEA). Reconstructed BEA can lead to loss of natural barrier function of sphincter of Oddi, predisposing to ascending cholangitis, with incidence reaching up to 10% (0%–47%) in patients with hepaticojejunostomy (HJ).^2^ Male sex, postoperative hepatolithiasis, and postoperative anastomotic strictures have been shown to be risk factors for cholangitis after BEA reconstruction.^2^ Up to 4.4% patients can develop recurrent cholangitis (median: 7 episodes) even with nonstenotic HJ.^3^ Overdevest et al reported overall incidence rate of was 21.5 episodes of nonobstructive recurrent cholangitis (NORC) per 100 person-years despite treatment.^3^ A step up approach for management of NORC has been proposed initially consisting of short course of antibiotics, followed by prolonged course of antibiotics, and third step consisting of biliary limb lengthening.^4^ Despite multiple steps, only 67% resolution rate of NORC has been reported.^4^ The most accepted hypothesis of NORC is reflux of food material into biliary tract through HJ due to lack of sphincter mechanism in reconstructed BEA.^5,6^ However, this hypothesis is not supported in the study by Overdevest et al, raising possibility of alternate underlying mechanisms of NORC in patients with hepato-pancreatobiliary (HPB) tract diversion.^3^

Microbiome alterations have been described after total pancreatectomy and islet autotransplant.^7^ The reflux of altered microbiome into nonstenosed HJ has been proposed as contributory mechanism of NORC.^1^ Authors reported complete resolution of NORC (5 episodes over 2 years) in patient with total pancreatectomy with auto islet transplantation and recurrent *Clostridioides difficile* infection after treatment with IMT. HPB tract diversion surgeries may also lead to changes in enterohepatic circulation and bile acid composition profile.^8,9^ Previous studies in patients with recurrent *C. difficile* suggest beneficial role of IMT in improving bile acid profile, which was subsequently associated with improved clinical outcomes.^10,11^ It has been hypothesized that bacteria plays a role in perpetuation of biliary inflammation in primary sclerosing cholangitis and previously a study demonstrated role of IMT engraftment in improving biochemical markers in this population.^12^

However, overall the implementation of IMT in individuals with surgically altered anatomy is not without its own challenges. The optimal route of delivery in individuals with HPB tract diversion is unknown. IMT can be utilized orally in the capsule form, nasojejunal/nasoduodenal tubes, enemas, or endoscopic/colonoscopic installation.^13^ Previous studies utilized capsule or nasoenteric route of delivery in patients with native intestinal anatomy and small intestinal bacterial overgrowth.^14,15^ Scott et al utilized simultaneous jejunal and colonoscopic delivery of IMT in the reported case.^1^ Limited data suggest that overall success does may differ by mode of delivery, and multiple routes of delivery may improve outcomes.^16,17^ The safety profile of IMT has not been well studied in patients with surgically altered anatomy. Marcella et al reported that overall serious adverse events occur in 1.4% individuals receiving IMT, with all being in patients with mucosal barrier injury.^18^ Overall rates of adverse events differ by mode of delivery, with upper gastrointestinal (GI) instillation associated with higher rates of serious adverse events, compared to lower GI route (1.4% vs 0.9%).^18^ Importantly, the AEs associated with upper GI instillation are mostly delivery-related AEs.^18^

In summary, HPB tract surgical diversion may lead to bacterial overgrowth and dysbiosis in the blind limb. “Restructuring” gut microbiome may be a potential therapeutic option for NORC, if future prospective studies show efficacy of IMT in this patient population. The optimal route of microbiome delivery and safety in patients with surgically altered anatomy should be investigated in future studies.

## DISCLOSURES

Author contributions: R. Karna conceptualization, drafting, editing, revision, and approval of final draft and is the article guarantor.

Financial disclosure: None to report.

Informed consent is not applicable here.
